# Migratory Songbirds as Potential Ectozoochorous Protist Dispersal Vectors

**DOI:** 10.1002/ece3.72703

**Published:** 2025-12-15

**Authors:** Silas E. Fischer, Joy J. Jackson, Elise C. Hoffman, Henry M. Streby, Trisha L. Spanbauer

**Affiliations:** ^1^ Department of Environmental Sciences The University of Toledo Toledo Ohio USA; ^2^ Earth and Environmental Sciences University of Kentucky Lexington Kentucky USA

**Keywords:** avian‐mediated dispersal, ectozoochory, epizoic diatoms, long‐distance dispersal, movement ecology, phoresy

## Abstract

Protist biogeography, speciation, and systematics continue to generate debate and inquiry because protist distributions and dispersal remain poorly resolved. Identifying potential vectors and basibionts for epibiont protists would contribute to our limited understanding of their ecology. Migratory animals seasonally link disparate landscapes, incidentally transporting other organisms in the process. Waterbirds are known microbe dispersers, but evidence for other avian groups is limited—such as taxa that often migrate longer distances. We asked whether terrestrial songbirds (Passeriformes) host diatoms (Bacillariophyta) by sampling tail plumage of four thrush species (Hermit Thrush *Catharus guttatus* , Swainson's Thrush *C. ustulatus*, Wood Thrush *Hylocichla mustelina* , American Robin *Turdus migratorius* ) spanning multiple migration strategies. Unexpectedly, we found diatoms in all seven samples (*n* = 4 tail snips, *n* = 3 tail swabs), yielding 224 individuals of 25 genera and 9 orders: primarily benthic, freshwater, raphe‐bearing genera (e.g., *Encyonopsis*, *Navicula*, *Nitzschia*). Several diatoms contained chloroplasts prior to digestion and slide mounting, consistent with potential viability. These natural history observations suggest that songbirds are overlooked carriers of hitchhiking diatoms, implying an undescribed but potentially important avian‐algal relationship.

## Graphical abstract

We demonstrate that migratory songbirds carry diatoms (Bacillariophyta) in their plumage, revealing an unexpected avian–algal association. All seven bird specimens we examined contained diatoms—224 frustules representing 25 genera and 9 orders, primarily freshwater, benthic pennates—some retaining chloroplasts prior to digestion and mounting (implying viability). Songbirds appear to be previously unknown basibiont hosts of hitchhiking diatoms, with the potential to facilitate long‐distance dispersal via migratory movements.

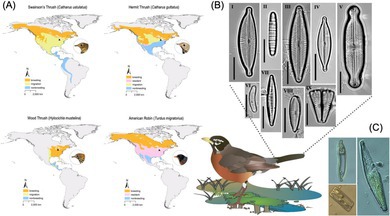

## 1 Introduction

Protist species distributions—including diatoms—have traditionally been described as cosmopolitan due to a lack of limitations on their dispersal abilities (i.e., the “ubiquity hypothesis”; Finlay et al. 2002), but recent work has demonstrated spatial population structure (Evans et al. 2009) and even endemism in some protists (i.e., “moderate endemicity”; Foissner et al. 2008; Kociolek et al. 2017; Pinseel et al. 2021). These observations, as well as accumulating evidence that many factors, such as historic processes and organismal traits, can shape the distributions of diatoms and other protists, suggest potential dispersal limitations (Mann and Vanormelingen 2013; Singer et al. 2019; Soininen and Teittinen 2019; Pinseel et al. 2024). However, dispersal mechanisms are still not well understood for most protists (e.g., diatoms, Keck et al. 2018; dinoflagellates, Tesson et al. 2018), and, accordingly, their biogeography, speciation processes, and systematics continue to generate debate and inquiry (Foissner 2006; Vanormelingen et al. 2008; Mann and Vanormelingen 2013; Soininen and Teittinen 2019; Pinseel et al. 2024). The debate is slowly moving beyond the question of whether protists are cosmopolitan or moderately endemic toward asking why, where, and which taxa may be more susceptible to dispersal limitation (Pinseel et al. 2024). Yet, our understanding of the mechanisms constraining protist dispersal and colonization remains incomplete—particularly regarding the identification and effectiveness of potential vectors.

Diatoms (Bacillariophyceae) are protists, more specifically highly speciose unicellular eukaryotic algae enclosed in siliceous cell walls that have captivated scientists and artists for centuries. These microalgae, which occur abundantly in both freshwater and marine systems, play a major role in primary productivity and are often used as bioindicators (Wehr et al. 2015). Some diatom species (and, in rare cases, genera) may be range‐restricted, but diatom families and higher taxonomic ranks are likely cosmopolitan in distribution (Vanormelingen et al. 2008; Pinseel et al. 2024). Diatoms are thought to disperse via water, wind, volcanic eruptions (Van Eaton et al. 2013), animals (Leone et al. 2014; Donato‐Rondón et al. 2018; Riaux‐Gobin et al. 2021), and by anthropogenic means (Kristiansen 1996), but the relative importance of these various mechanisms is not known. Animal‐mediated transport, in particular, may be a regularly occurring means of diatom dispersal, especially by migratory animals such as waterfowl (Bahls 2025). Further, some diatoms are epizoic, living as surface‐dwelling epibionts on their host organisms, or basibionts (e.g., Bennett 1920; Majewska and Goosen 2020). Although there is a small body of literature documenting epizoic diatom phenomena and an even smaller subset suggesting animal‐mediated diatom dispersal, most studies focus on aquatic animals (e.g., sea turtles—Donato‐Rondón et al. 2018; Riaux‐Gobin et al. 2021; fish—Shin et al. 2004; waterbirds—Croll and Holmes 1982; Manning et al. 2021; whales—Bennett 1920), with few examples from terrestrial species.

Migratory animals connect disparate landscapes across the planet through seasonal annual cycle movements. Thus, migratory animals can act as short‐ and long‐distance dispersal vectors (Viana et al. 2016; Mogle et al. 2018) through both endozoochory (i.e., transport of ingested material) and ectozoochory (i.e., transport of external material; sometimes called exo‐ or epi‐zoochory) of dispersal units (Figuerola and Green 2002; Lewis et al. 2014; Green et al. 2023), including propagules (e.g., seeds and spores; Warner and French 1970; Viana et al. 2012; Chmielewski and Eppley 2019; Martín‐Vélez et al. 2022), individual organisms such as diatoms (Leone et al. 2014; Coughlan et al. 2017), and even invasive species (Russo et al. 2019). Among animals, migratory birds may play the largest role as dispersal vectors because they traverse significant geographical barriers and often make stopovers (i.e., resting and refueling sites) along their migratory routes, enabling propagule deposition and attachment (Viana et al. 2012, 2016). Considering roughly one in five bird species globally is thought to be migratory, there is enormous potential for avian‐mediated microbe dispersal—which Darwin (1859) hypothesized almost two centuries ago in aquatic plants, seeds, and other dispersal units.

Here, we use natural history‐rooted microscopy observations of swabs and feather tissue from songbird specimens to explore the potential for a novel, previously overlooked animal‐alga association. We collected thrushes (Passeriformes: Turdidae) that had collided with windows on a college campus—a common cause of anthropogenic avian mortality (Loss et al. 2014; Fischer and Islam 2020)—followed by sampling their feathers, digesting organic material present, and preparing slides with reflective mountant to highlight the siliceous diatom frustules. We performed microscopy transects to record all instances of diatom presence and report that all seven samples contained diatoms. Interestingly, we identified 224 individual diatom frustules of 25 genera across 9 orders, primarily freshwater, benthic pennates (e.g., *Encyonopsis*, *Navicula*, *Nitzschia*), some of which contained chloroplasts prior to digestion and mounting. Songbirds appear to be previously unknown basibiont hosts of hitchhiking epizoic diatoms, with potential to facilitate dispersal via directed, migratory movements.

## 2 Methods

**FIGURE 1:**
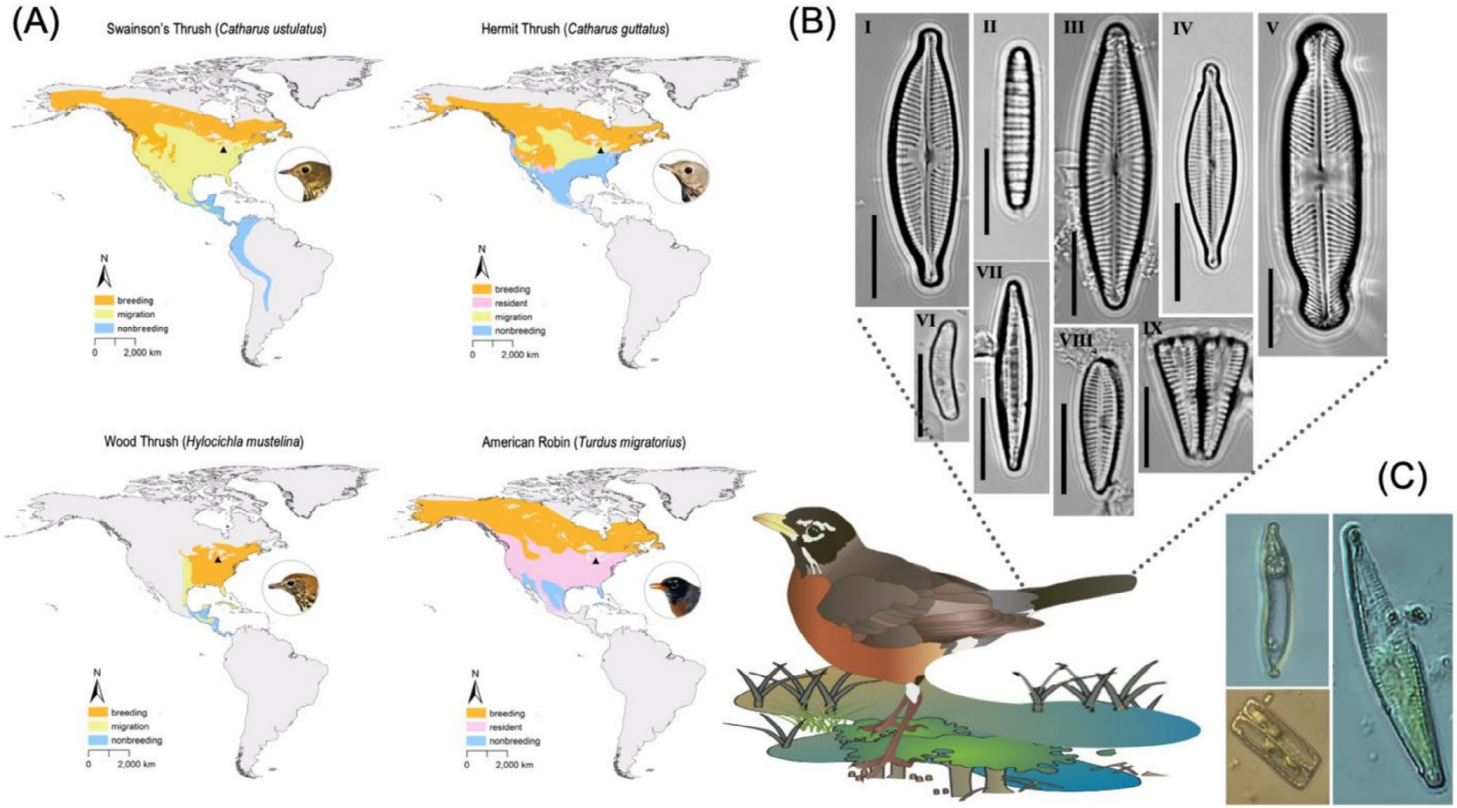
Annual cycle range maps for the 4 thrush species (Family Turdidae) sampled, indicating potential diatom attachment and deposition locations (A). Black triangles denote collection location (The University of Toledo campus, Toledo, OH, USA). Examples of diatoms found on American Robin ( *Turdus migratorius* ) tail swab and tail snip (B). I = *Cymbopleura*, II = *Diatoma*, III = *Navicula*, IV = *Encyonopsis*, V = *Pinnularia*, VI = *Eunotia*, VII = *Nitzschia*, VIII = *Gomphonema*, and IX = girdle view of unidentified pennate diatom. All scale bars = 10 μm. Examples of pennate diatoms with chloroplasts photographed during initial microscopy (i.e., prior to sample digestion and slide mounting) at ×400 (C). Range map (A) information is adapted from BirdLife International (2021; datazone.birdlife.org/species/requestdis). Thrush photos (A) modified and reproduced with permission from Andrea Lindsay, courtesy of Powdermill Nature Reserve, Carnegie Museum of Natural History. American Robin and wetland graphics (B) by Tracey Saxby, Integration and Application Network (ian.umces.edu/media‐library). Microscopy photographs by Silas E. Fischer (C) and Joy J. Jackson (B).

We screened songbird tails for diatoms in November 2022 as an exploratory investigation of potential terrestrial bird‐mediated ectozoochorous dispersal of diatoms. We sampled four individual thrush specimens of four species collected in 2019 during peak songbird migration as fresh window‐collision mortalities—a common anthropogenic cause of avian mortality—on The University of Toledo campus (Toledo, OH, USA; Table 1). Bird species included American Robin ( *Turdus migratorius* ), Hermit Thrush ( *Catharus guttatus* ), Swainson's Thrush ( *C. ustulatus* ), and Wood Thrush ( *Hylocichla mustelina* ), representing multiple migratory strategies from facultative, short‐distance to obligate, long‐distance (Table 1, Figure 1A). Notably, Swainson's Thrush and Hermit Thrush only migrate through the region; they do not breed or overwinter near the collection location. Wood Thrushes do breed in OH but not on the university campus. The nonbreeding range for Hermit Thrush includes the southern USA, Mexico, Guatemala, and Bermuda (Dellinger et al. 2020). The nonbreeding range for Wood Thrush includes southern Mexico and most of Central America (Stanley et al. 2014), whereas Swainson's Thrush nonbreeding range extends from southern Mexico to northern Argentina (Mack and Yong 2020). We therefore assumed that these three species were migrants. Our understanding of American Robin migration is currently limited; some populations are facultative migrants, sometimes making large movements, and others apparently do not migrate (Jahn et al. 2019). Thus, we do not know the migration status of the American Robin we sampled, and we acknowledge it is possible that the individual bred locally.

**TABLE 1:** Sample types, identifiable diatom genera (in alphabetical order), and valve counts from examined slides (*n* = 7 total) from tails of four individuals of four thrush species (Passeriformes: Turdidae) collected as window collision mortalities on The University of Toledo campus (OH, USA) in 2019 during migration. Thrush migration strategies were obtained from the AVONET database (https://doi.org/10.6084/m9.figshare.16586228.v7).

Bird species	American Robin ( *Turdus migratorius* )		Hermit Thrush ( *Catharus guttatus* )		Swainson's Thrush^a^ ( *Catharus ustulatus* )	Wood Thrush ( *Hylocichla mustelina* )	
Migration strategy	Facultative short‐distance		Obligate long‐distance		Obligate long‐distance	Obligate long‐distance	
Collection date	8 Apr 2019		16 Apr 2019		24 Sep 2019	2 May 2019	
Sample type	Tail snip	Tail swab	Tail snip	Tail swab	Tail snip	Tail snip	Tail swab
Diatom genus							
*Achnanthes*	—	—	—	—	1	—	—
*Achnanthidium*	—	—	—	1	—	—	2
*Amphora*	—	—	—	—	—	—	1
*Aulacoseira*	—	—	—	1	—	—	—
*Caloneis*	—	—	1	—	—	—	—
*Cocconeis*	—	1	—	—	—	1	—
*Cymbopleura*	2	—	—	—	—	—	—
*Diatoma*	—	1	2	2	3	3	—
*Diploneis*	—	—	—	1	—	—	—
*Encyonema*	1	—	—	—	—	—	2
*Encyonopsis*	12	9	9	9	8	17	3
*Eunotia*	8	—	—	2	1	—	—
*Gomphonema*	3	1	4	4	2	5	1
*Hantzschia*	2	—	—	—	1	—	1
*Luticola*	—	1	—	—	—	—	—
*Meridion*	—	—	—	1	1	1	—
*Navicula*	7	5	4	4	5	4	1
*Nitzschia*	2	—	—	3	5	6	7
*Pinnularia*	1	—	—	2	4	1	2
*Planothidium*	2	—	—	—	—	—	1
*Prestauroneis*	—	—	—	—	1	—	—
*Rhoicosphenia*	—	—	—	—	—	1	—
*Sellaphora*	2	—	1	—	1	1	—
*Stephanocyclus*	—	—	—	—	—	1	—
*Surirella*	2	2	2	6	5	1	—

^a^ No tail swab sample collected.

**FIGURE 2:**
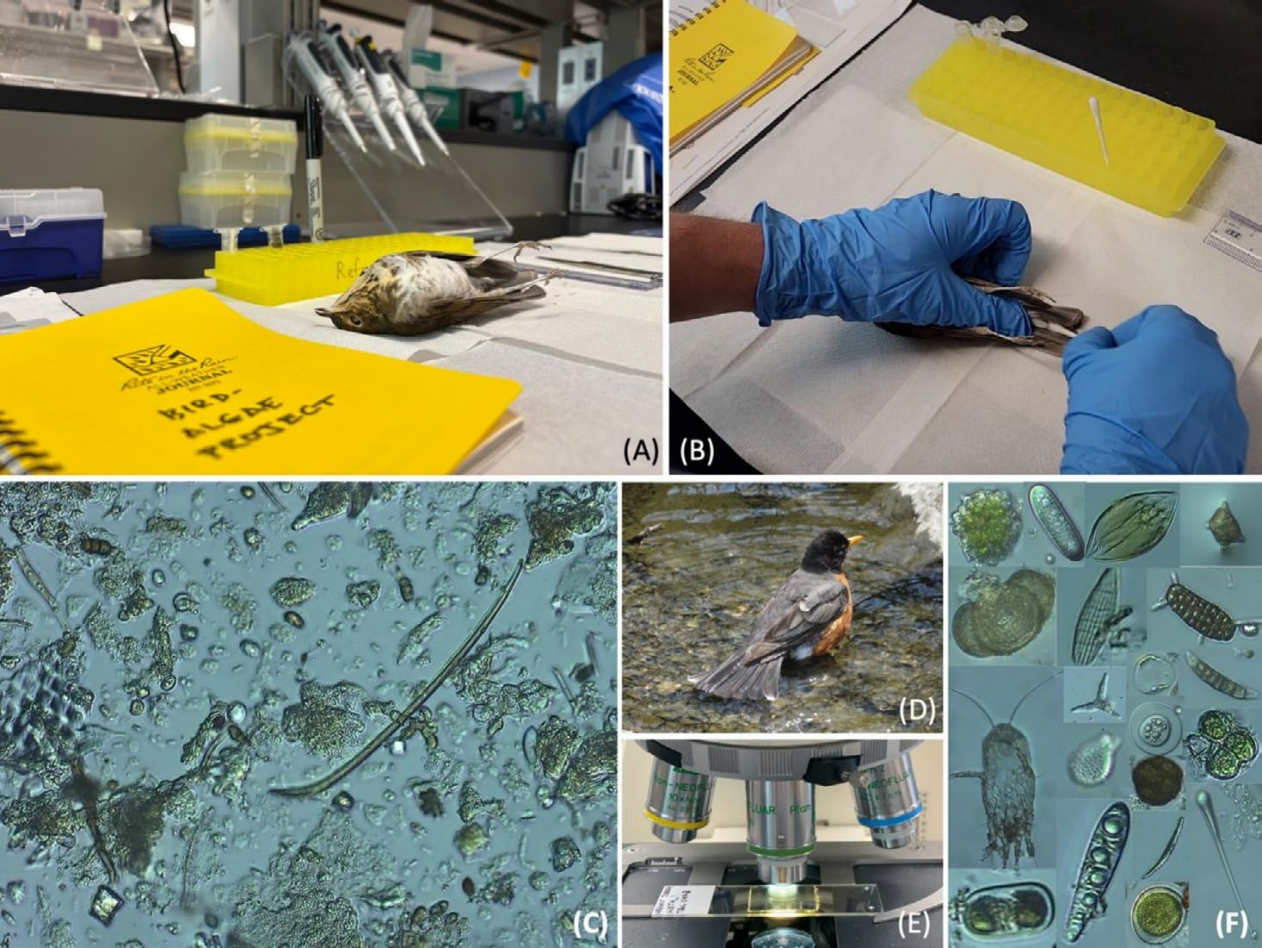
Swabbing a Swainson's Thrush ( *Catharus ustulatus* ) tail (A, B). Example images of organisms, propagules, and other material and structures from initial microscopy (i.e., prior to sample digestion and slide mounting; C, F). Note that the images are not to scale and were photographed at ×200 and ×400 (C, F). An American Robin ( *Turdus migratorius* ) foraging and/or bathing in a stream (D; photo by SK Winnicki with permission). Microscopy of a mounted slide to perform diatom transects (E).

Briefly, we followed methods described by Johansson et al. (2021) to collect tail feather snips and swabs from the frozen bird specimens collected under USFWS Wildlife Permit MB09838B (Figure 2A,B). We snipped ~2 cm from one inner rectrix tail feather (R1 or R2) and then used cotton swabs to remove material from tail feathers (Johansson et al. 2021; Figure 2A,B). Samples were sonicated at 40 kHz (FS20D, Fisher Scientific) and centrifuged at 16,000 *g* (for detailed methods, see Johansson et al. 2021). Swabs and feathers were then removed while leaving the “pellet” of organic material in the bottom of the tube and centrifuged again for 3 min at 16,000 *g*. We then removed most of the supernatant, leaving only a “pellet” and a small amount of water for examination (Johansson et al. 2021).

We initially examined samples under ×200–×400 magnification via light microscopy (Zeiss Axioplan 2) and then digested each sample in 30% H_2_O_2_. Samples were transferred to 22 × 22 mm coverslips, air‐dried, and mounted on slides with Naphrax (PhycoTech Inc., MI, USA). We identified, to genus level, all slide‐mounted diatoms in transects at ×200–×400 magnification and photographed each diatom at ×1000 magnification with DIC optics (Zeiss Axioscope 5; Figure 2E). We referenced Lange‐Bertalot et al. (2017) and Spaulding et al. (2021) for diatom genus identification via morphology (e.g., valve shape and symmetry, striae pattern). All data are available on Figshare (Fischer et al. 2025).

## 3 Results

**FIGURE 3:**
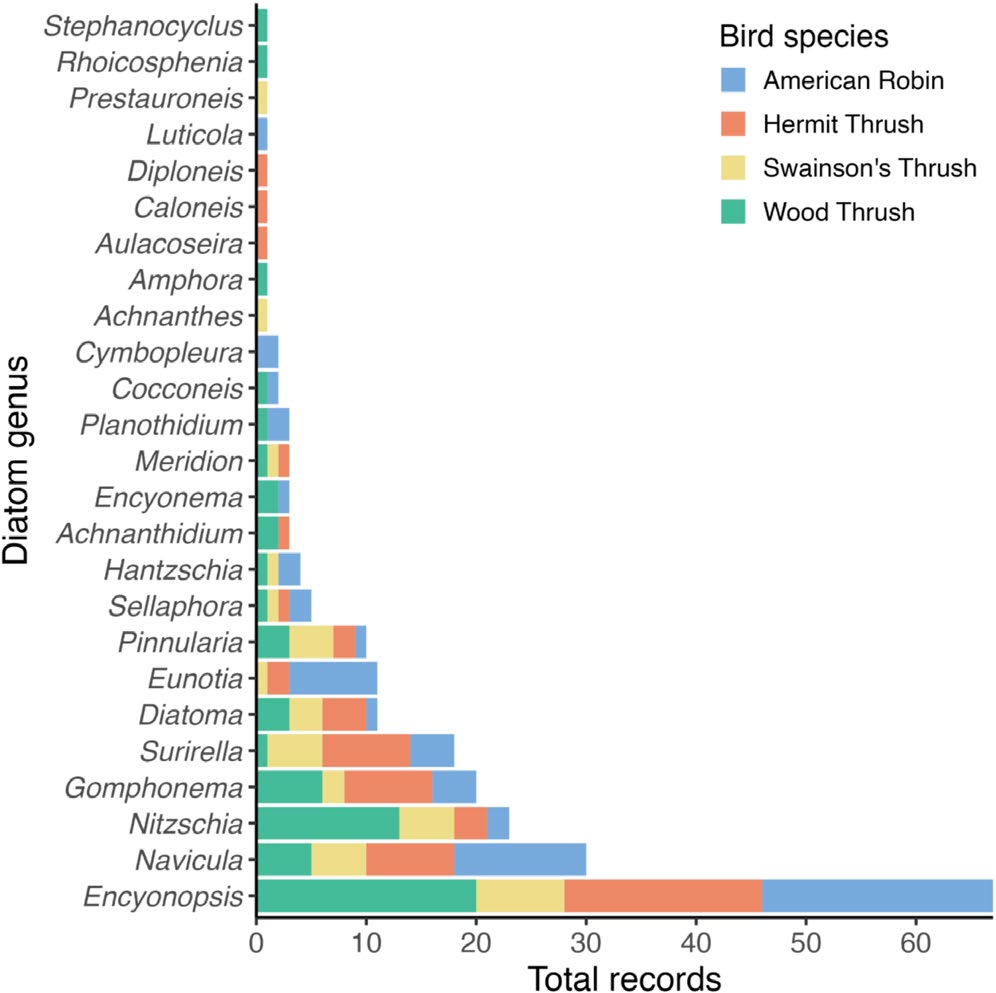
Number of individual diatom frustules of each genus across all seven slides of the four thrush species (Passeriformes: Turdidae) sampled: American Robin ( *Turdus migratorius* ), Hermit Thrush ( *Catharus guttatus* ), Swainson's Thrush ( *C. ustulatus* ), and Wood Thrush ( *Hylocichla mustelina* ).

We found diatoms attached to thrush tail feathers from all seven slides we examined (*n* = 4 tail snips, *n* = 3 tail swabs; *n* = 4 individual bird specimens; Table 1, Figure 1B). Diatoms present were primarily benthic, freshwater, raphe‐bearing genera (Figure 1B). Notably, some diatoms contained chloroplasts prior to digestion and mounting (e.g., Figure 1C). We identified 224 individual diatoms of 25 genera overall, of which the top five genera were *Encyonopsis*, *Navicula*, *Nitzschia*, *Gomphonema*, and *Surirella* (full list in dataset; Table 1, Figure 3). These 25 genera spanned 9 orders, including Achnanthales, Aulacoseirales, Bacillariales, Cymbellales, Eunotiales, Fragilariales, Naviculales, Surirellales, and Thalassiophysales. Among the four thrush species and two sample types, the number of genera present per sample ranged from 7 to 13. *Encyonopsis*, *Diatoma*, *Gomphonema*, *Navicula*, *Nitzschia*, *Pinnularia*, *Sellaphora*, and *Surirella* were present in all seven samples. Other propagules and organisms we found during initial microscopy (i.e., prior to digestion and slide mounting for diatom transects) included but were not limited to pollen grains, free‐living green algae, freshwater sponge spicules, phytoliths, feather mites, fungal spores, and other miscellaneous diaspores (Figure 2C,F).

## 4 Discussion

We found an unexpected diversity and abundance of freshwater diatoms attached to songbird tail feathers, with observations of > 200 diatom frustules of 25 genera and 9 orders. The four thrush species we sampled (all of which hosted diatoms in their plumage) exhibit diverse migration strategies in terms of timing and distance. Additionally, we found live chloroplasts in several diatoms prior to digestion (Figure 1C). We contend that there is compelling potential for short‐ and long‐distance avian‐mediated diatom dispersal, but validation is required to confirm this hypothesis. Given our observations, and that species interactions are a major shortfall to our knowledge of biodiversity (i.e., the Eltonian shortfall; Hortal et al. 2015), this avenue of research warrants greater attention.

Though our observations do not provide direct evidence of diatom dispersal per se (Johansson et al. 2021), the abundance of frustules and the presence of intact chloroplasts in some frustules (e.g., see Figure 1C) are consistent with viability at the time we collected the birds as recent window collision mortalities (i.e., before freezing). Intact organelles and photosynthetic pigments are reasonable indications of recent viability, and these morphological indicators are often used to distinguish live or recently living diatoms from empty frustules (e.g., Peterson 1987; Barral‐Fraga et al. 2018); nevertheless, these observations are not conclusive evidence of viability or dispersal and should not be interpreted as such. However, songbirds and other closely related birds (e.g., woodpeckers [Piciformes]) have recently been shown to transport and disperse other protists and propagules (Lewis et al. 2014; Chmielewski and Eppley 2019; Johansson et al. 2021, 2025), with some evidence of avian movements shaping disjunct distributions of, e.g., bryophytes (Coughlan et al. 2015). Even if bird‐mediated diatom dispersal were uncommon relative to other dispersal modes, rare dispersal events can still shape species distributions (Popp et al. 2011; Chmielewski and Eppley 2019). Diatom species distributions are poorly constrained, and range maps are limited for most diatom species and genera, as in many protists. The limited existing knowledge of diatom distributions is scattered, regionally restricted, or taxonomically incomplete, and basic taxonomy is unresolved in many cases as well (Vanormelingen et al. 2008). Moreover, most diatom genera are thought to be cosmopolitan, precluding the ability to provide more rigid support for dispersal here.

Birds have long been known to host and transport algae (Proctor 1959; Schlichting Jr. 1960), including diatoms (Atkinson 1972; Sides 1973; Croll and Holmes 1982), but previous work has largely focused on endozoochory by examining bird gut contents and feces rather than ectozoochorous transport (Figuerola and Green 2002; Coughlan et al. 2017). Further, most studies on bird‐mediated endozoochory (e.g., Schlichting Jr. 1960; Atkinson 1972; Sides 1973) and ectozoochory (e.g., Sides 1973; Croll and Holmes 1982; Manning et al. 2021) focus on waterbirds, such as gulls and ducks (Figuerola and Green 2002; Green et al. 2023). Bahls (2025) posited that disjunct distributions of some diatoms may be explained by waterfowl movements between flyways. However, empirical ectozoochorous studies are scant (Costa et al. 2014), especially in terrestrial avian taxa.

Johansson et al. (2021) screened the plumage and feet of three forest‐dwelling woodpeckers and found algae (including diatoms, e.g., *Meridion circulare* and *Pinnularia* spp.) and other propagules. The woodpeckers were not associated with water (Johansson et al. 2021), suggesting vector potential in unsuspecting terrestrial species. Our observations of diatoms and other microbes in songbird plumage align with those of Johansson et al. (2021), Chmielewski and Eppley (2019), and Warner and French (1970). Importantly, many songbirds, including most of the Nearctic–Neotropical migratory thrushes in our study, can migrate longer distances and are often more abundant compared to the avian taxa in which diatoms have previously been found, highlighting potential for longer‐distance algal dispersal than previously described. For example, Swainson's Thrushes breed as far north as the forests of the Arctic Circle and migrate to nonbreeding sites as far south as Argentina (Figure 1A), making stopovers along the way in a variety of cover types (e.g., coastal areas, riparian corridors), many of which host water sources. During such stopovers (and throughout their annual cycle), thrushes and other songbirds often interface with water sources while foraging or bathing, allowing potential attachment of diatoms (e.g., Figure 2D). For example, Slessers (1970) described bathing behavior in landbirds, noting that thrushes sometimes enter “ecstatic” or “purgatorial” bathing stages in which they submerge in water “…so enthusiastically the body becomes a mass of disheveled watersoaked feathers…” (see figure 2 in Slessers 1970). Although it is not apparent where or when the diatoms were attached, these four thrush species also often forage on the ground, gleaning arthropods from soil and leaf litter (Sabo 1980; Holmes and Robinson 1988), making diatom attachment plausible.

The most common diatom we observed was *Encyonopsis*, a genus of freshwater diatoms found primarily in oxygen‐rich waters of mountains and higher latitudes as well as in the tropics (Krammer 1997). These conditions are consistent with thrush habitat associations across the annual cycle, implying overlap. We found *Encyonopsis* among all seven samples, totaling 67 records, or roughly 30% of the diatoms we observed. There are records of *Encyonopsis* attached to other animals, such as crayfish (Falasco et al. 2018), Wels catfish (*Siluris glanis*; Falasco et al. 2025), and green sea turtles ( *Chelonia mydas* ; Pennesi et al. 2023).

Diatom species traits, such as cell size and substrate attachment ability, may play a role in explaining our observations of primarily small benthic diatoms present in our samples and, more broadly, patterns of diatom biogeography, especially freshwater diatom taxa. Small, benthic species dominate diatom assemblages on sea turtles (Riaux‐Gobin et al. 2021), consistent with our observations. Survival and viability of diatoms and other propagules on songbirds, as for most other animal vectors, are unknown (Coughlan et al. 2015). However, Manning et al. (2021) experimentally demonstrated that diatoms (i.e., *Nitzschia pusilla* ) adhering to waterfowl ( *Anas platyrhynchos* ) plumage can remain viable during flight. Leone et al. (2014) estimated that one diatom species ( *Didymosphenia geminata* ) can remain viable on mink ( *Neovison vison* ) fur for ~60d. Flight time, temperature, and humidity are important factors to diatom desiccation and viability (Souffreau et al. 2013; Manning et al. 2021), and low moisture levels are likely common in freshwater diatom dispersal events (Souffreau et al. 2013). Unlike other protists, diatoms are encased in silica frustules, which may slow desiccation and promote viability. Several aspects of songbird plumage and ecology likely encourage diatom attachment. Songbird feathers, like those of waterfowl, may act as humid, insulating microclimates and may slow desiccation in addition to providing ample surface area and topography for diatom attachment (Coughlan et al. 2015; Manning et al. 2021), especially if contained within mud or biofilms on the external surface of a bird. We recommend that future research focuses on experimentally investigating and/or modeling the desiccation tolerances and viability of diatoms while attached to songbirds and other animals during and across different stages of the annual cycle, both in the lab and in the field, to provide insight into potential dispersal processes (Viana et al. 2013; Manning et al. 2021). Culturing diatoms sampled from external surfaces of birds would also contribute to understanding their viability (e.g., Ashworth et al. 2022). Host species traits and behavior may influence their likelihood and effectiveness as vectors (Chmielewski and Eppley 2019; Riaux‐Gobin et al. 2021; Ashworth et al. 2022), which merits further study.

We identified diatoms via microscopy and morphology; future work would be most effective combined with molecular approaches (Soininen and Teittinen 2019; Pérez‐Burillo et al. 2025; but see Riaux‐Gobin et al. 2021; Ashworth et al. 2022). Where possible, species‐level diatom identification may provide insight into where attachment occurred, particularly if any of the diatom species occupy putatively limited distributions that may aid in identifying specific bird locations throughout the annual cycle (critical information for enacting targeted avian conservation actions). Recent work has suggested similar potential in combining palynology and ornithology (Goodenough and Webb 2025). We suggest sampling for diatoms and other dispersal units on both live birds and collected carcasses as a low‐cost, non‐invasive addition to existing projects or as a use of existing natural history museum collections (e.g., Kanjer et al. 2020; Johansson et al. 2021). In particular, integrative studies that combine movement tracking technology (e.g., geolocators and GPS tags) with propagule sampling (e.g., Prakash et al. 2022) and/or diatom‐based bioindication indices could reveal patterns of avian migratory connectivity as it relates to habitat quality, dispersal ecology, and diatom biogeography over ecological and evolutionary time scales.

## 5 Conclusion

Our study provides new insight into unexplored vertebrate‐alga interactions. These observations warrant further investigation into diatom viability and potential for avian‐mediated dispersal dynamics. Given the ubiquity of both diatoms and birds, and the enormous volume of songbirds that migrate biannually between geographically disparate landscapes across the globe, songbird‐mediated dispersal could be a potential factor shaping the distributions and genetic connectivity of microorganisms such as diatoms.

## Author Contributions

**Silas E. Fischer:** conceptualization (equal), data curation (lead), formal analysis (lead), funding acquisition (equal), investigation (equal), methodology (equal), visualization (lead), writing – original draft (lead), writing – review and editing (lead). **Joy J. Jackson:** investigation (equal), methodology (equal), validation (lead), visualization (supporting), writing – original draft (supporting), writing – review and editing (supporting). **Elise C. Hoffman:** funding acquisition (equal), investigation (supporting), writing – original draft (supporting), writing – review and editing (supporting). **Henry M. Streby:** investigation (supporting), project administration (supporting), resources (equal), supervision (supporting), writing – original draft (supporting), writing – review and editing (supporting). **Trisha L. Spanbauer:** conceptualization (equal), formal analysis (supporting), funding acquisition (equal), investigation (supporting), methodology (lead), project administration (lead), resources (equal), supervision (lead), validation (supporting), visualization (supporting), writing – original draft (supporting), writing – review and editing (supporting).

## Funding

This work was supported by the University of Toledo, USR‐CAP.

## Conflicts of Interest

The authors declare no conflicts of interest.

## Data Availability

All data are available on Figshare (https://doi.org/10.6084/m9.figshare.24295582).
